# CLL, together with C1qR, suppresses WSSV infection by regulating the activation of Dorsal

**DOI:** 10.1128/jvi.00416-25

**Published:** 2025-10-13

**Authors:** Xiao-Tong Cao, Lian-Jie Wu, Jia-Yu Si, Xian-Wei Wang, Jiang-Feng Lan

**Affiliations:** 1 Shandong Provincial Key Laboratory of Animal Biotechnology and Disease Control and Prevention, College of Veterinary Medicine, Shandong Agricultural University 34734, Taian, China; 2 Shandong Provincial Key Laboratory of Animal Cells and Developmental Biology, School of Life Sciences, Shandong University, Qingdao, Shandong, China; Wageningen University & Research, Wageningen, Netherlands

**Keywords:** white spot syndrome virus, *Procambarus clarkii*, lectin, Dorsal, C1q receptor

## Abstract

There is no evidence that arthropods possess a complement system. In arthropods, only gC1qR has been reported. The functional convergence of gC1qR in arthropods and vertebrates during evolution remains to be further elucidated. In the present study, a C-type lectin named *Pc*CLL, which contains a C1q-like sequence (a segment of the C1q globular head) and might be involved in antiviral immunity, was identified in *Procambarus clarkii*. When crayfish are infected with white spot syndrome virus (WSSV), some C1qRs (gC1qR homologs), which are located at the cell membrane, recognize WSSV by interacting with the envelope protein VP28. Subsequently, C1qR recruits *Pc*CLL in the cell, and the C1qR-*Pc*CLL complexes synergistically promote the nuclear translocation of Dorsal. Serine-arginine protein kinase (SRPK) is an essential molecule in this process. The C1qR*-Pc*CLL-SRPK axis regulates the activation and translocation of Dorsal. Finally, the C1qR*-Pc*CLL-Dorsal axis can promote resistance to WSSV infection by regulating the expression of the lectin-like protein (*Pc*LT). In both vertebrates and arthropods, C1qR appears to participate in antiviral immunity, but the mechanisms were different. This may highlight a fascinating evolutionary trajectory across animals.

**IMPORTANCE:**

The body’s recognition of pathogenic microorganisms plays an important role in host resistance to pathogen infection. Here, we identified a lectin, *Pc*CLL, in the red swamp crayfish *Procambarus clarkii*, which contains a C1q-like sequence and might be involved in antiviral immunity. C1qR located on the cell membrane can recognize white spot syndrome virus by interacting with VP28. Then, it recruits *Pc*CLL and promotes the nuclear translocation of Dorsal. This process cannot be achieved without SRPK, which is a splicing regulator that can phosphorylate proteins. CLL (C1q-like motif that is part of the globular head of C1q) and C1qR (gC1qR homolog) interact with each other, and they are involved in the antiviral immune response by activating Dorsal. This may be a new mechanism for the host to recognize pathogens.

## INTRODUCTION

The complement system is one of the oldest branches of the immune system and plays an important role in both innate and adaptive immunity in vertebrates. The complement system is composed of more than 30 soluble and membrane-bound proteins, a group of precisely regulated protein response systems widely present in serum and tissue fluid and on the surface of the cell membrane (1–3). C1q is one of the initiating components of the complement system. C1q mediates host immune defense against pathogenic microorganisms by recognizing antibody-antigen complexes or pathogen surface molecules to trigger a complement cascade (4–6). C1q also interacts with the receptor for the globular heads (gC1qR), and this interaction mediates immune responses, including phagocytosis, inflammation, the uptake of apoptotic cells, and infection (7–9). Because gC1qR and C1q also recognize and bind pathogens (such as HIV, hepatitis B virus, hepatitis C virus, pneumococci), many pathogens exploit the normal function of C1q/C1qR to escape host immunity (7, 10–12). Pneumococcal endopeptidase O (PepO) promotes bacterial adherence and attenuates the complement pathway by interacting with C1q (12). The C1q/C1qR-mediated complement system plays an important role in various life processes. However, the complement system has not been reported in arthropods.

Unlike vertebrates, invertebrates (including arthropods) can rely only on innate immunity to resist pathogenic invasion (13, 14). Only a few complement-related molecules have been identified in invertebrates, including thioester protein (iTEP) in insects (15), the ficolin homolog AsFCN in sea squirts (16), and *Cg*CLec-CCP, *Cg*C3, and C3-like receptors in oysters (17, 18). However, in arthropods, only C1qR has been found (19–22). In *Macrobrachium rosenbergii*, *Mr*gC1qR participates in the immune response by recognizing pathogen-associated molecular patterns (such as lipopolysaccharide [LPS] and peptidoglycan [PGN]) (21). In *Scylla paramamosain*, *Sp*gC1qR restricted white spot syndrome virus (WSSV) replication by interacting with the envelope protein VP28 of WSSV (23). However, whether arthropods have a mechanism for pathogen recognition similar to C1q/gC1qR remains unclear.

In this study, we identified a C-type lectin (*Pc*CLL) with a C1q-like sequence from crayfish. *Pc*CLL can be recruited by C1qR and is involved in the anti-WSSV immune response. This study helps elucidate the evolution of the complement system and the function of lectin in the antipathogen immunity of crayfish.

## RESULTS

### *Pc*CLL inhibits the replication and infection of WSSV in red swamp crayfish

**Fig 1:**
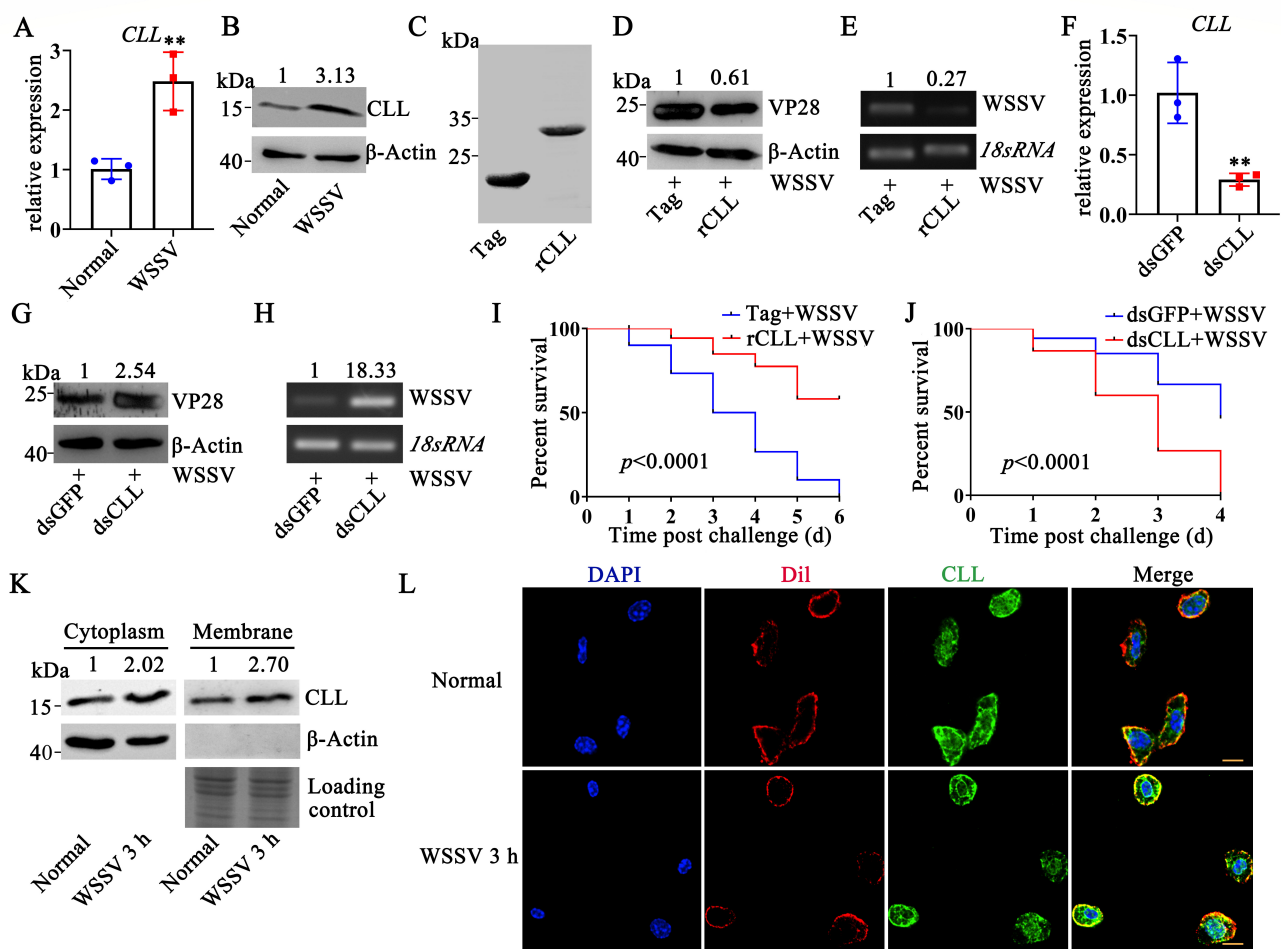
*Pc*CLL responds to WSSV challenge and suppresses WSSV infection. (**A, B**) The expression pattern of *Pc*CLL after WSSV infection was analyzed by qRT-PCR and Western blotting. 18S RNA and β-actin were used as controls. (**C**) Purified His-Tag and His-*Pc*CLL proteins. (**D, E**) Effect of rCLL administration *in vivo* on WSSV infection was detected. Crayfish were injected with rCLL or the control Tag and then infected with WSSV 2 h later. VP28 levels (**D**) and the relative value of WSSV copies (**E**) were detected at 24 h post-WSSV infection by Western blotting and semiquantitative PCR, respectively. (**F**) The efficiency of *Pc*CLL-RNAi was determined at 48 h after dsCLL injection. DsGFP was used as a control. (**G, H**) Effect of CLL RNAi on WSSV infection was detected. WSSV infection was performed at 48 h post-dsRNA injection. VP28 levels (**G**) and the relative value of WSSV copies (**H**) were detected another 24 h later by Western blotting and semiquantitative PCR, respectively. (**I**) Effect of rCLL application on crayfish survival after WSSV infection was analyzed. Crayfish (*n* = 30) were injected with rCLL or the control Tag and then infected with WSSV. The survival rate was recorded. (**J**) The survival rate was determined after WSSV infection in dsCLL-injected crayfish (30 crayfish each group). (**K**) The subcellular location of *Pc*CLL was detected by Western blotting. (**L**) Confocal fluorescence microscopy was used to determine the distribution of *Pc*CLL on the cell membrane. DAPI (blue) marks the nucleus, Dil (red) marks the cell membrane, and green shows *Pc*CLL. Scale bar = 5 µm. Differences among the groups were analyzed using a *t*-test, and the asterisk represents a significant difference, **, *P* < 0.01 (Student’s *t*-test). Repeats were performed in triplicate with at least three crayfish for each sample.

C-type lectins reportedly perform multiple functions in antiviral immunity and participate in immune responses through recognition, adhesion, and killing of pathogens. Twenty-one C-type lectins were identified from the gill transcriptome after WSSV infection, and the expression levels of eight genes were significantly increased. Structural analysis of the amino acid (AA) sequences of these eight genes was performed using SMART. A C-type lectin with a C1q-like sequence, named *Pc*CLL, was found in red swamp crayfish. As shown in Fig. S1, *Pc*CLL is 2,396 bp in length, containing a 501 bp open reading frame that encodes 166 AA. The predicted molecular weight of the protein is approximately 18 kDa. *Pc*CLL contains a CLECT domain, which includes a C1q-like motif of 61 AA. Additionally, *Pc*CLL lacks a signal peptide. To study the role of *Pc*CLL in antiviral immunity, quantitative real-time polymerase chain reaction (qRT-PCR) and Western blot assays were performed to determine the expression profile of *Pc*CLL. The transcription level of *Pc*CLL in WSSV-infected crayfish was 2.45-fold of that in normal crayfish, and its protein level was 3.13-fold of that in normal crayfish (Fig. 1A and B). The recombinant protein of *Pc*CLL (rCLL) was obtained (Fig. 1C) and administered to crayfish hemocoels to generate an overexpression-like effect. WSSV inoculation was then performed in rCLL-injected crayfish to determine whether rCLL would influence viral infection. rCLL application significantly decreased the levels of VP28 (the most abundant envelope protein of WSSV) (Fig. 1D) and the relative value of WSSV copies (Fig. 1E). Moreover, rCLL application suppressed the mortality induced by WSSV infection (Fig. 1I), suggesting that *Pc*CLL might inhibit WSSV infection. To verify the results, RNA interference (RNAi) was performed to silence *Pc*CLL expression. As shown in Fig. 1F, the expression of *Pc*CLL was significantly downregulated by double-stranded RNA (dsRNA) application. WSSV infection was then performed in dsCLL-treated crayfish to determine whether *Pc*CLL interference would influence viral infection. The results showed that decreased expression of *Pc*CLL led to an increase in VP28 expression (Fig. 1G) and the relative value of WSSV copies (Fig. 1H), as well as a decrease in survival rate (Fig. 1J). To further investigate the function of *Pc*CLL, we analyzed the subcellular localization of *Pc*CLL after WSSV infection. Compared with normal crayfish, *Pc*CLL levels were significantly increased both in the cell cytoplasm (2.02-fold) and on the cell membrane (2.7-fold, Fig. 1K) 3 h after WSSV infection. An immunofluorescence staining assay via confocal microscope showed that CLL was colocalized with the cell membrane (Fig. 1L). These results suggest that *Pc*CLL inhibits WSSV infection.

### *Pc*CLL interacts with C1qR

**Fig 2:**
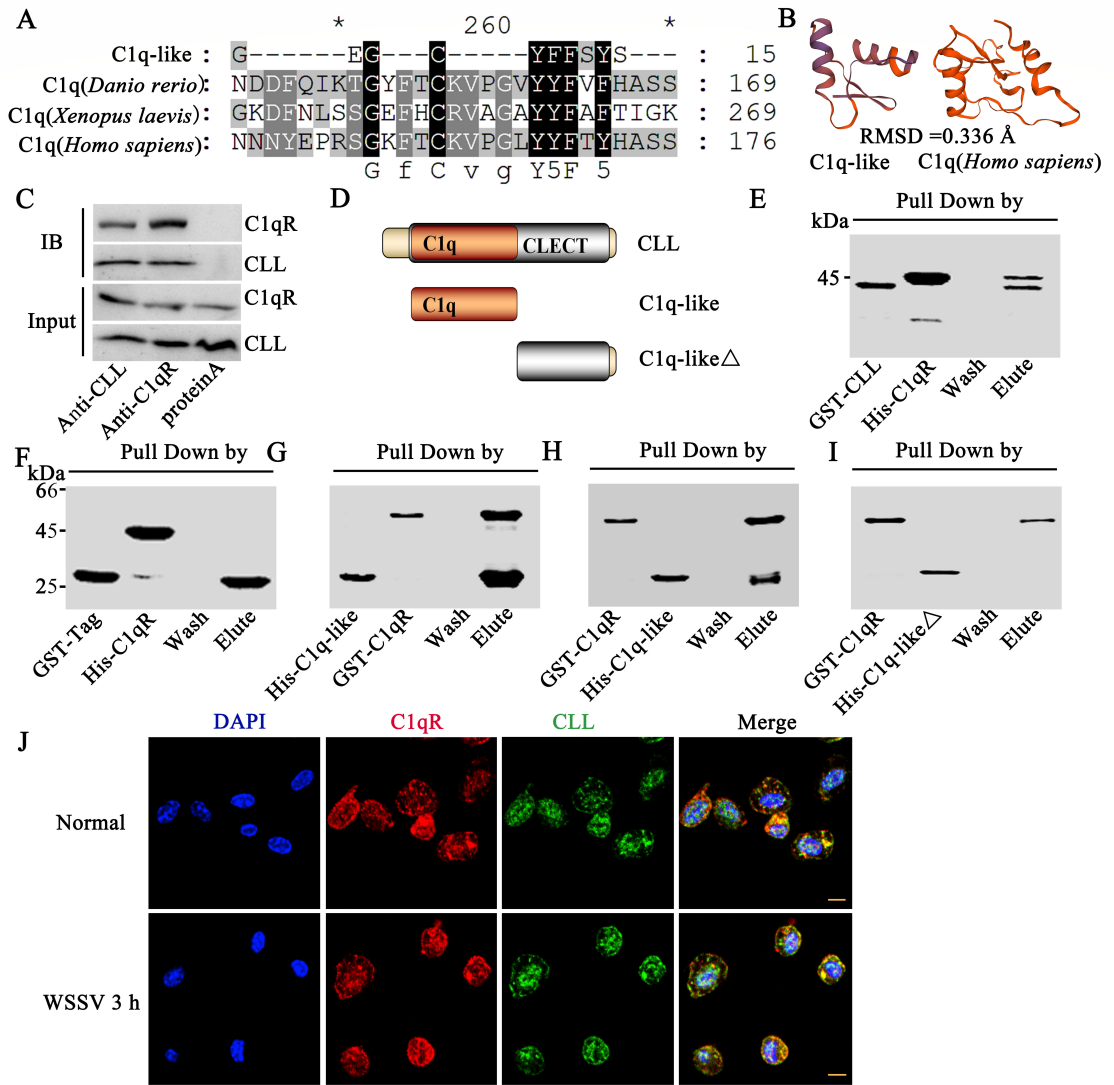
*Pc*CLL interacts with C1qR. (**A**) Multiple alignments of the C1q-like region of *Pc*CLL with C1qs (globular head) (*Danio rerio*, *Xenopus laevis,* and *Homo sapiens*) were performed using MEGA 6 and GeneDoc software. (**B**) The structures of the C1q-like region of *Pc*CLL and C1q domain of *Hs*C1q (*H. sapiens*) were analyzed using the online SWISS-MODEL server, and their RMSD was analyzed using PyMOL software. (**C**) Interactions between *Pc*CLL and C1qR were determined using a co-IP assay. (**D**) *Pc*CLL fragments were segmented as *Pc*CLL, C1q-like, and C1q-likeΔ. (**E**) Determination of the interactions of rCLL and rC1qR by pull-down assays. rGST-CLL was incubated with GST resin, and then, rHis-C1qR was added. RGST-CLL and its interacting proteins were bound to the GST resin. The GST resin was eluted, and the samples were analyzed by SDS-PAGE (stained with Coomassie Brilliant Blue). (**F**) GST-Tag was used as a control to verify the interaction of rGST-CLL and rHis-C1qR. (**G**) Determination of interactions of rC1q-like and rC1qR by pull-down assays. rHis-C1q-like was incubated with His resin, and then rGST-C1qR was added. His resin was used to extract the rHis-C1q-like protein and the interacting proteins. The His resin was eluted, and the samples were analyzed by SDS-PAGE (stained with Coomassie Brilliant Blue). (**H**) Determination of the interactions of rGST-C1qR and rHis-C1q-like by pull-down assays. rGST-C1qR was incubated with GST resin, and then rHis-C1q-like was added. RGST-C1qR and its interacting proteins were bound to the GST resin. The GST resin was eluted, and the samples were analyzed by SDS-PAGE (stained with Coomassie Brilliant Blue). (**I**) Determination of the interactions of rGST-C1qR and rHis-C1q-likeΔ by pull-down assays. rGST-C1qR was incubated with GST resin, and then rHis-C1q-likeΔ was added. The GST resin was eluted, and the samples were analyzed by SDS-PAGE (stained with Coomassie Brilliant Blue). (**J**) Detection of the subcellular colocalization of *Pc*CLL and C1qR by confocal fluorescence microscopy. DAPI (blue) marks the nucleus, red shows C1qR, and green shows *Pc*CLL. Scale bar = 5 µm. IB, immunoprecipitates were detected with the antibody specific for each panel.

SMART (http://smart.embl.de/) analysis revealed that *Pc*CLL contains a C1q-like motif that is part of the globular head of the C1q C-terminus (Fig. S1B). Multiple alignment analysis of the amino acid sequences of four C1q/C1q-like motifs (globular heads) (*Procambarus clarkii* with 61 AA, *Danio rerio* with 138 AA, *Xenopus laevis* with 144 AA, and *Homo sapiens* with 136 AA) was performed using MEGA 6 and GeneDoc software, which revealed conserved areas (Fig. 2A). The three-dimensional structures of the C1q-like region of *Pc*CLL (from crayfish, 61 AA) and C1q domain of *Hs*C1q (from *H. sapiens*, 136 AA) were subsequently predicted using the online SWISS-MODEL server. These two domains exhibited similar α-helix structures, with a root mean square deviation (RMSD) of 0.336 Å (calculated via PyMOL software, Fig. 2B). According to previous reports, the globular head of C1qs can interact with gC1qRs. We analyzed whether *Pc*CLL interacts with C1qR (gC1qR homolog) by a coimmunoprecipitation (co-IP) assay. The results suggest that *Pc*CLL can interact with *Pc*C1qR (Fig. 2C). To further detect the interaction of C1q-like and C1qR, we obtained recombinant proteins of the full-length *Pc*CLL, C1q-like, and C1q-likeΔ strains (Fig. 2D). A pull-down assay was used to verify the interaction, and the results revealed that *Pc*CLL and C1q-like could interact with C1qR (Fig. 2E through H), whereas C1q-likeΔ could not interact with C1qR (Fig. 2I). An immunofluorescence chemistry assay via confocal microscope was subsequently used to analyze the colocalization of *Pc*CLL and C1qR, and the colocalization of *Pc*CLL and C1qR was observed (Fig. 2J). In summary, it can be concluded that *Pc*CLL colocalizes and interacts with C1qR.

### C1qR inhibits WSSV replication by binding the WSSV envelope protein VP28

**Fig 3:**
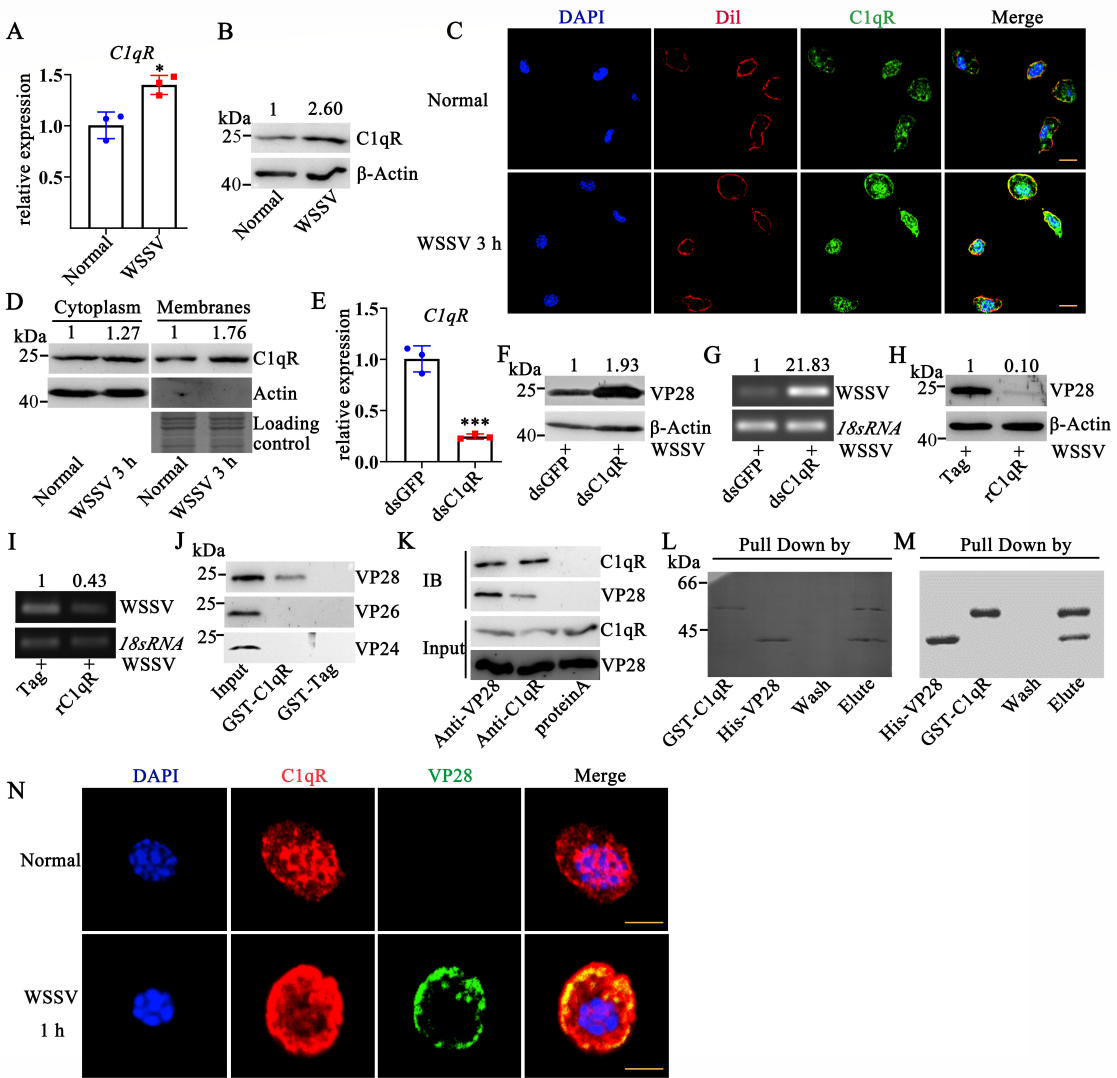
C1qR is involved in antiviral immunity and recognizes WSSV by interacting with VP28. (**A, B**) Expression patterns of C1qR after WSSV infection were analyzed by qRT-PCR and Western blotting. 18S RNA and β-actin were used as controls. (**C**) Confocal fluorescence microscopy was used to detect the subcellular localization of C1qR and the membrane. DAPI (blue) marks the nucleus, Dil (red) marks the cell membrane, and green shows C1qR. (**D**) The subcellular localization of C1qR was detected by Western blotting through the analysis of cytoplasmic and membrane protein fractions. (**E**) The efficiency of C1qR-RNAi was determined at 48 h after dsC1qR injection. DsGFP was used as a control. (**F, G**) Expression levels of VP28 (**F**) and the relative value of WSSV copies (**G**) were analyzed by Western blotting and semiquantitative PCR after dsC1qR and WSSV injection. (**H, I**) Expression levels of VP28 (**H**) and the relative value of WSSV copies (**I**) were analyzed by Western blotting and semiquantitative PCR after rC1qR and WSSV injection. (**J**) Screening of viral proteins that interact with C1qR using pull-down and Western blot assays. (**K**) Interaction of C1qR with VP28 was determined using a co-IP assay. (**L, M**) Analysis of the interaction of rGST-C1qR with rHis-VP28 by a pull-down assay. (**N**) Detection of the subcellular colocalization of C1qR and VP28 (WSSV) by confocal fluorescence microscopy. DAPI (blue) marks the nucleus, red shows C1qR, and green shows VP28 (WSSV). Scale bar = 5 µm. Differences among the groups were analyzed using a *t*-test, and the asterisk represents a significant difference; *, *P* < 0.05; ***, *P* < 0.001 (Student’s *t*-test). IB, immunoprecipitates were detected with the antibody specific for each panel. Repeats were performed in triplicate with at least three crayfish for each sample.

The role of C1qR in anti-WSSV immunity remains to be further studied. When crayfish were infected with WSSV, C1qR expression was induced at the mRNA level (1.43-fold, Fig. 3A) and at the protein level (2.6-fold, Fig. 3B). And the protein level of C1qR was increased both in the cell cytoplasm (1.27-fold) and on the cell membrane (1.76-fold, Fig. 3D) after WSSV infection. Immunofluorescence staining via confocal microscope was used to detect the localization of C1qR on the membrane after WSSV infection. As shown in Fig. 3C, C1qR colocalized on the membrane with Dil, a cell membrane marker. To further study the inhibitory role of C1qR in WSSV infection, C1qR was silenced with RNAi (0.23-fold, Fig. 3E). In C1qR-RNAi crayfish, the expression levels of VP28 (1.93-fold, Fig. 3F) and the relative level of WSSV copies (21.83-fold, Fig. 3G) increased after 24 h WSSV infection. Significant reductions in VP28 expression levels and the relative level of WSSV copies were observed in rC1qR-injected crayfish after WSSV infection (Fig. 3H and I). These results indicate that C1qR might inhibit WSSV replication or infection. In addition to identifying pathogens by binding to immunoglobulin, C1q can also recognize pathogens directly and then interact with C1qR (4, 5, 8). Previous studies have reported that gC1qR also has a strong affinity for pathogenic microorganisms (7, 11, 23). The WSSV envelope protein plays an important role in viral infection. Pull-down and Western blotting assays were used to analyze the interaction between C1qR (gC1qR homolog) and major envelope proteins. As shown in Fig. 3J, C1qR could interact with VP28 but not with VP24 or VP26. We further studied the interaction between C1qR and VP28 by using co-IP and pull-down assays. As shown in Fig. 3K, native C1qR could interact with native VP28. GST-C1qR could bind to His-VP28 (Fig. 3L), and His-VP28 could bind to GST-C1qR (Fig. 3M). The above results suggest that C1qR might inhibit the replication or infection of WSSV by binding to VP28. To analyze whether the interaction between C1qR and VP28 occurs in the early stage of infection, immunofluorescence staining via confocal microscope was used to evaluate the colocalization of C1qR and VP28 at an early stage after WSSV injection. The colocalization of VP28 (WSSV) and naive C1qR at 1 h after WSSV infection is shown in Fig. 3N. These results suggest that C1qR might sense WSSV infection by recognizing VP28 in the early stage of WSSV infection.

### *Pc*CLL and C1qR promote Dorsal translocation into the nucleus

**Fig 4:**
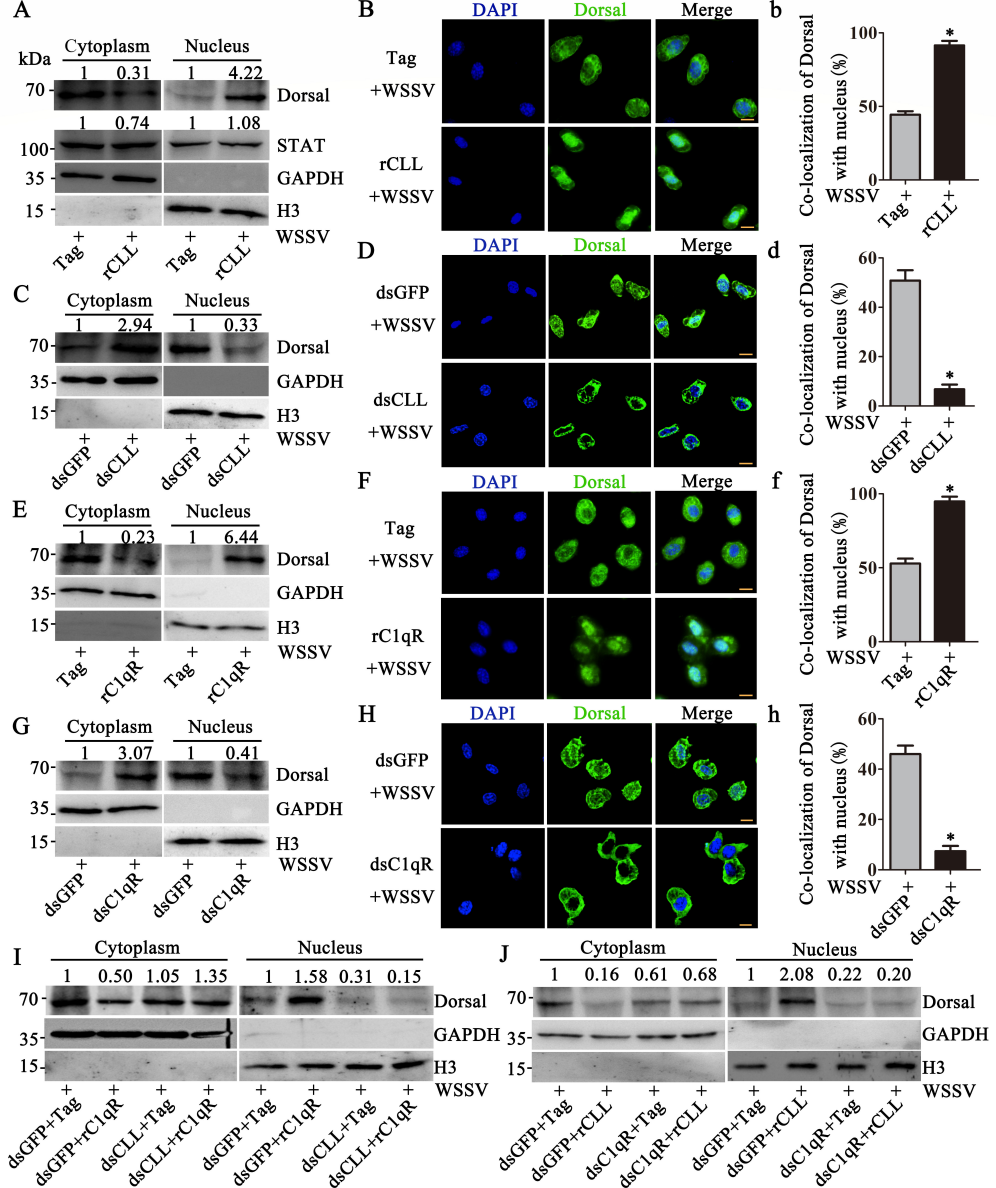
*Pc*CLL and C1qR induce Dorsal translocation into the nucleus from the cytoplasm. (**A**) The nuclear translocations of Dorsal and STAT were detected by Western blotting after WSSV infection in rCLL-injected crayfish. (**B**) Immunofluorescence staining was performed using confocal fluorescence microscopy to detect the colocalization of Dorsal with the nucleus in rCLL-treated crayfish hemocytes. RTag was used as a control. (**b**) Statistical analysis of the colocalization between Dorsal and the nucleus was performed using WCIF ImageJ software. (**C**) The nuclear translocation of Dorsal was detected by Western blotting after WSSV infection in dsCLL-injected crayfish. (**D**) Immunofluorescence staining was performed using confocal fluorescence microscopy to detect the colocalization of Dorsal with the nucleus in dsCLL-treated crayfish hemocytes. DsGFP was used as a control. (**d**) Statistical analysis of **D**. (**E**) The nuclear translocation of Dorsal was detected by Western blotting after WSSV infection in rC1qR-injected crayfish. (**F**) Dorsal translocation into the nucleus in hemocytes was evaluated in rC1qR-injected crayfish. (**f**) Statistical analysis of **F**. (**G**) The nuclear translocation of Dorsal was detected by Western blotting after WSSV infection in dsC1qR-injected crayfish. (**H**) Dorsal translocation in the hemocytes of C1qR-RNAi crayfish was detected using confocal fluorescence microscopy. (**h**) Statistical analysis of **H**. (**I**) The subcellular distribution of Dorsal was detected by Western blotting after WSSV infection in dsCLL-RNAi and rC1qR-injected crayfish. (**J**) The subcellular distribution of Dorsal was detected by Western blotting after WSSV infection in dsC1qR- and rCLL-injected crayfish. Scale bar = 5 µm. Differences among the groups were analyzed using a *t*-test, and the asterisk represents a significant difference, *P* < 0.05. Repeats were performed in triplicate with at least three crayfish for each sample.

The NF-κB and JAK/STAT signaling pathways play important roles in anti-WSSV immunity (24–26). WSSV infection was performed at 2 h post-rCLL application. By separating and analyzing the cytoplasmic and nuclear proteins after 6 h of WSSV infection, a significant nuclear shift in Dorsal (4.22-fold compared to Tag-injected crayfish) was detected in rCLL-injected crayfish, while no changes in STAT nuclear translocation were detected (Fig. 4A). This finding was further supported by immunofluorescence staining via confocal microscope (Fig. 4B). The proportion of Dorsal located in the nucleus increased from 44% to 91% after rCLL injection (Fig. 4b). WSSV infection was performed at 48 h post-dsCLL injection, and the subcellular localization of Dorsal was analyzed in these dsCLL-injected crayfish after 6 h of WSSV infection. In dsCLL-injected crayfish, Dorsal levels in the nucleus decreased (0.33-fold compared with dsGFP-injected crayfish, Fig. 4C), and the proportion of Dorsal in the nuclear was decreased from 50% to 7% (Fig. 4D and d). Similarly, injection of rC1qR promoted Dorsal nuclear translocation after 6 h of WSSV infection (6.44-fold compared with Tag-injected crayfish, Fig. 4E), and the transfer ratio increased from 53% to 94% (Fig. 4F and f). C1qR-RNAi inhibited Dorsal nuclear translocation (0.41-fold compared with dsGFP-injected crayfish, Fig. 4G), and the transfer ratio was reduced from 46% to 8% (Fig. 4H and h). We also silenced the expression of *Pc*CLL or C1qR separately: after silencing *Pc*CLL, we injected rC1qR; after silencing C1qR, we injected rCLL. Subsequently, the crayfish were infected with WSSV to determine whether *Pc*CLL and C1qR synergistically promote the nuclear translocation of Dorsal. We found that C1qR injection promoted Dorsal nuclear translocation (1.58-fold, Fig. 4I). Injection of rC1qR after *Pc*CLL-RNAi did not promote Dorsal nuclear translocation (0.15-fold, Fig. 4I). *Pc*CLL injection promoted Dorsal nuclear translocation (2.08-fold, Fig. 4J). Injection of rCLL after C1qR-RNAi in crayfish did not promote Dorsal nuclear translocation (0.20-fold, Fig. 4J). Therefore, these results demonstrated that *Pc*CLL and C1qR cooperate to promote the nuclear translocation of Dorsal.

### *Pc*CLL- and C1qR-induced Dorsal translocation depends on SRPK

**Fig 5:**
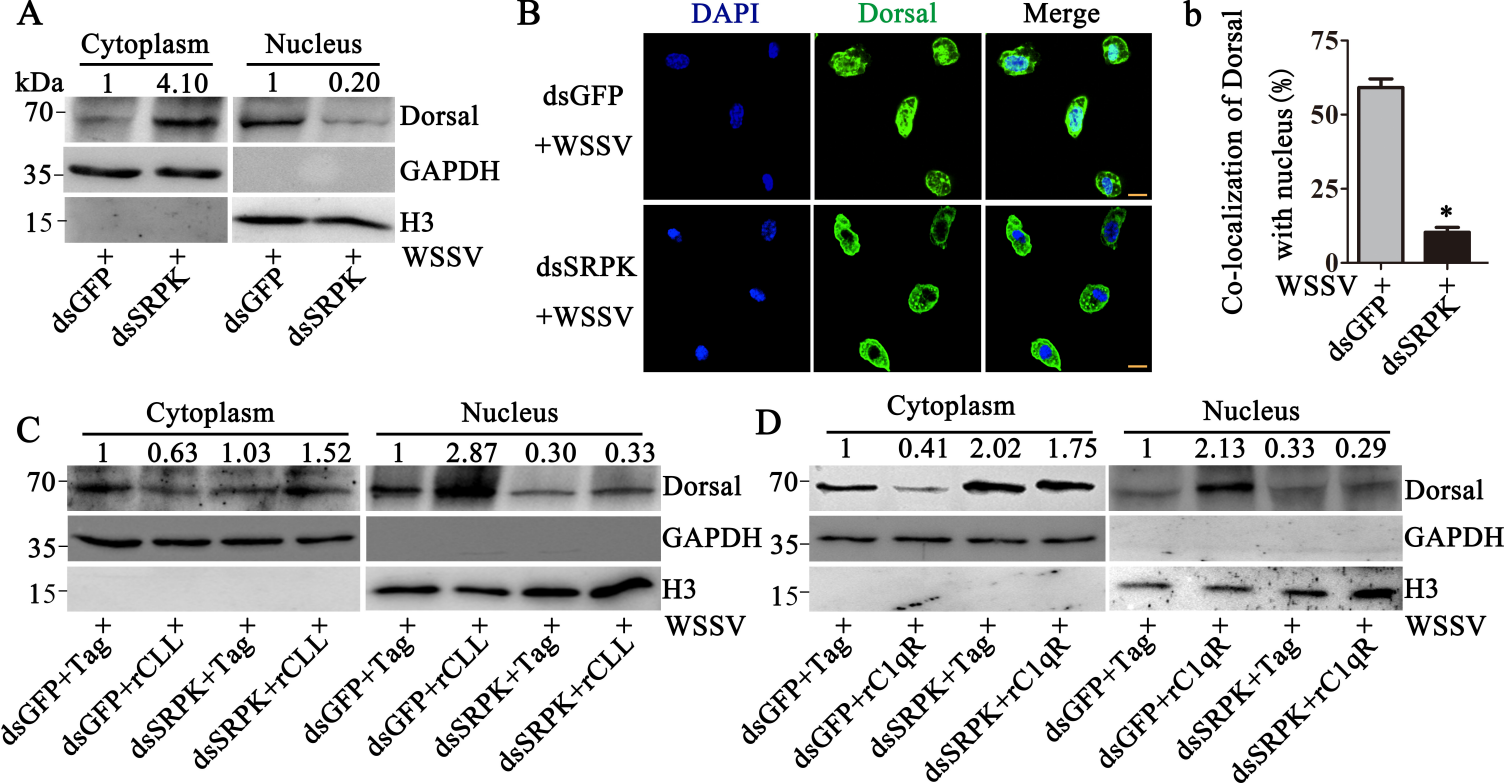
Dorsal transfer from the cytoplasm into the nucleus is dependent on SRPK. (**A**) The subcellular distribution of Dorsal was detected by Western blotting after WSSV infection in dsSRPK-injected crayfish. DsGFP was used as a control. (**B**) Dorsal translocation was detected using confocal fluorescence microscopy in the hemocytes of SRPK-RNAi-treated crayfish after WSSV infection. (**b**) Statistical analysis of the data in **B**. (**C**) Effect of SRPK-RNAi on rCLL-enhanced nuclear translocation of Dorsal was detected by Western blotting. Injection of rCLL and WSSV was performed at 48 h post-dsRNA injection. The nuclear translocation of Dorsal was detected at 6 h post-WSSV infection. (**D**) Effect of SRPK-RNAi on rC1qR-enhanced nuclear translocation of Dorsal was detected by Western blotting. Injection of rC1qR and WSSV was performed at 48 h post-dsRNA injection. The nuclear translocation of Dorsal was detected at 6 h post-WSSV infection. Colocalization was analyzed by WCIF ImageJ software. Scale bar = 5 µm. Differences among the groups were analyzed using a *t*-test, and the asterisk represents a significant difference, *P* < 0.05. Repeats were performed in triplicate with at least three crayfish for each sample.

Most transcription factors are modified in the cytoplasm and then enter the nucleus to initiate the transcription of immune effector molecules (27, 28). To further investigate the antiviral immune function of *Pc*CLL, we reviewed many studies and discovered a serine-arginine protein kinase (SRPK), which functions as a splicing regulator (29), phosphorylates a variety of proteins (30), and is involved in the phosphorylation of transcription factors in multiple signaling pathways (including the NF-κB pathway) (31, 32). The role of SRPK during Dorsal activation was analyzed. To determine whether interfering with SRPK expression would influence Dorsal nuclear translocation, WSSV was administered to crayfish hemocoels at 48 h post-dsSRPK injection. The results suggested that the proportion of Dorsal that entered the nucleus decreased (0.2-fold of that in dsGFP-injected crayfish, Fig. 5A), and the transfer ratio decreased from 59% to 10% (Fig. 5B and b) in dsSRPK-injected crayfish. To further analyze whether the *Pc*CLL- and C1qR-mediated regulation of Dorsal depends on SRPK, dsSRPK was injected, followed by separate injection of rCLL or rC1qR. Subsequently, the crayfish were infected with WSSV. We found that silencing of SRPK inhibited the nuclear translocation of Dorsal. The results showed that *Pc*CLL and C1qR regulate the activation of Dorsal in an SRPK-dependent manner (Fig. 5C and D).

### *Pc*CLL and C1qR inhibit WSSV infection by regulating the expression of *Pc*LT

**Fig 6:**
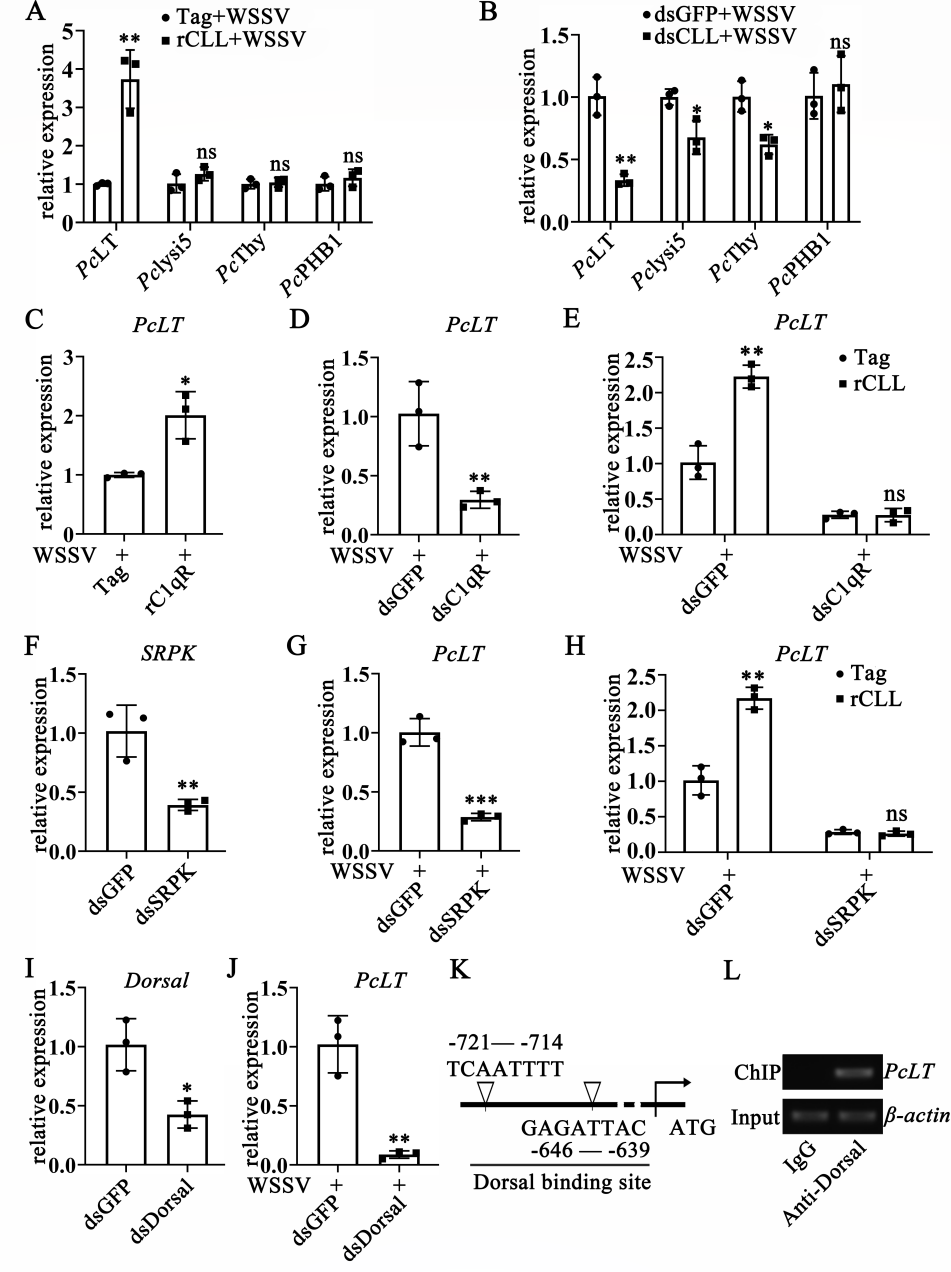
The *Pc*CLL-C1qR-SRPK-Dorsal axis regulates the expression of *Pc*LT. (**A**) Effect of rCLL application on the levels of *Pc*LT, *Pc*lysi5, *Pc*Thy, and *Pc*PHB1 was detected by qRT-PCR. Crayfish were infected with WSSV at 2 h after first injection of rCLL. The expression level of *Pc*LT was detected at 6 h after WSSV infection. (**B**) Effect of *Pc*CLL-RNAi on the transcription levels of *Pc*LT, *Pc*lysi5, *Pc*Thy, and *Pc*PHB1 was determined by qRT-PCR after WSSV infection in dsCLL- or dsGFP-injected crayfish. (**C**) Effect of rC1qR application on the level of *Pc*LT was detected by qRT-PCR. Crayfish were infected with WSSV at 2 h after first injection of rC1qR. The expression level of *Pc*LT was detected at 6 h after WSSV infection. (**D**) The transcription level of *Pc*LT was measured after WSSV infection in dsC1qR-injected crayfish. (**E**) The effect of C1qR-RNAi on the enhancement of *Pc*LT expression by *Pc*CLL after WSSV infection was detected by qRT-PCR. Application of rCLL and WSSV was performed at 48 h post-dsRNA injection. The expression level of *Pc*LT was detected 6 h later. (**F**) RNAi efficiency of SRPK was determined by qRT-PCR in dsSRPK-injected crayfish. (**G**) Effect of SRPK-RNAi on the level of *Pc*LT was detected. The expression level of *Pc*LT was analyzed by qRT-PCR after WSSV infection in dsSRPK-injected crayfish. (**H**) The effect of SRPK-RNAi on the enhancement of *Pc*LT expression by *Pc*CLL after WSSV infection was detected by qRT-PCR. Application of rCLL and WSSV was performed at 48 h post-dsRNA injection. The expression level of *Pc*LT was detected 6 h later. (**I**) RNAi efficiency of Dorsal was determined by qRT-PCR in dsSRPK-injected crayfish. (**J**) Effect of Dorsal RNAi on the level of *Pc*LT was detected by qRT-PCR. (**K**) Analysis of the *Pc*LT promoter. The 5´ untranslated region of *Pc*LT was analyzed by the online Promoter Scan tool. (**L**) ChIP together with RT-PCR was used to determine Dorsal binding to the promoter of *Pc*LT. Differences among the groups were analyzed using a *t*-test, and the asterisk represents a significant difference; *, *P* < 0.05; **, *P* < 0.01; ***, *P* < 0.001 (Student’s *t*-test). Repeats were performed in triplicate with at least three crayfish for each sample.

After Dorsal enters the nucleus, it participates in the antiviral immune response by regulating the transcription of antimicrobial peptides or antiviral protein genes (33). WSSV was administered to crayfish hemocoels at 2 h post-rCLL application. After 6 h of WSSV infection, we analyzed four molecules (*Pc*LT, *Pc*Lysi5, *Pc*Thy, and *Pc*PHB1) reported to participate in the anti-WSSV immune response by qPCR (34–37). The results showed that the transcription level of *Pc*LT was increased in rCLL- and WSSV-injected crayfish; however, infection with WSSV in rCLL-injected crayfish did not affect the transcription levels of *Pc*Lysi5, *Pc*Thy, and *Pc*PHB1 (Fig. 6A). To further analyze the effects of *Pc*CLL on the transcript levels of four antiviral genes, WSSV infection was performed on crayfish at 48 h post-dsCLL injection, and the transcription of four antiviral genes was analyzed 6 h after WSSV infection. In dsCLL-injected crayfish, the transcription levels of *Pc*LT (0.33-fold), *Pc*Lysi5 (0.67-fold), and *Pc*Thy (0.62-fold) were decreased (Fig. 6B) compared with those in dsGFP-injected crayfish. On the basis of the above results, we confirmed that *Pc*CLL could regulate the transcription of *Pc*LT. Similarly, the transcription level of *Pc*LT increased in rC1qR-injected crayfish compared with that in control Tag-injected crayfish (2.01-fold, Fig. 6C). The transcription level of *Pc*LT decreased after C1qR-RNAi (0.29-fold, Fig. 6D) compared with that in dsGFP-injected crayfish. The transcription level of *Pc*LT could be induced by rCLL injection after green fluorescent protein (GFP)-RNAi (2.23-fold, Fig. 6E), and the transcription level of *Pc*LT did not change significantly after rCLL injection under C1qR-RNAi (Fig. 6E). After SRPK-RNAi (Fig. 6F) and Dorsal RNAi (Fig. 6I) *in vivo*, we analyzed the transcription level of *Pc*LT and found that SRPK-RNAi or Dorsal-RNAi injection reduced the transcription level of *Pc*LT following WSSV infection (Fig. 6G and J). The transcription level of *Pc*LT could be induced by rCLL injection after GFP-RNAi (2.17-fold, Fig. 6H), and the transcription levels of *Pc*LT did not change significantly after *Pc*CLL injection under SRPK-RNAi (Fig. 6H). These results show that *Pc*CLL, C1qR, SRPK, and Dorsal can regulate the expression level of *Pc*LT. To further confirm this conclusion, the online Promoter Scan tool was used to analyze whether the 5′ untranslated region of *Pc*LT contains a Dorsal binding site. Two Dorsal binding sites were found (−721 to −714 and −646 to −639) in the 5′ untranslated region of *Pc*LT (Fig. 6K). Chromatin immunoprecipitation (ChIP) was used to confirm the binding of Dorsal to the identified sites (Fig. 6L). These data suggest that *Pc*CLL, C1qR, and Dorsal can regulate the expression of *Pc*LT in an SRPK-dependent manner.

## DISCUSSION

The complement system, which is involved in opsonic and lytic effector pathways, is thought to exist only in vertebrates (38, 39). Recent reports have shown that in invertebrates, oyster C-type lectin (*Cg*CLec), with a complement control protein domain, activates the complement system by interacting with mannose-binding lectin-associated serine protease (*Cg*MASPL) to promote *Cg*C3 cleavage (17). Four proteins with a C1q domain were identified in *Hyriopsis cumingii* (40). However, there are no reports of complement systems in arthropods. Currently, only C1qR has been reported in arthropods. Lectins may play different roles in the immune response. Reports have shown that lectins, in addition to the CRD domain, may also contain other special domains to perform different functions. In our previous study, *Mj*CC-CL was shown to interact with Domeless via its IL-like sequence and then be involved in antibacterial immunity by inducing the phosphorylation of STAT in kuruma shrimp (41). In the present study, we identified a lectin with a C1q-like sequence, named *Pc*CLL, which was found to interact with *Pc*C1qR (Fig. 2). It is involved in the anti-WSSV immune response in crayfish.

The globular heads of C1q interact with gC1qR (the receptor for the globular heads of C1q) (7–9). gC1qR in crustaceans (such as *Portunus trituberculatus*, *M. rosenbergii*, *S. paramamosain*) contains a mitochondrial acidic matrix protein (MAM33), which is similar to that of vertebrates (19, 21, 23). *Penaeus monodon* C1q subcomponent binding protein (*Pm*C1qBP) has been reported to interact with C1q in mice (42). The amino acid sequence of C1qR from crayfish was analyzed online by SMART, and it was shown that C1qR contains a MAM33 domain, indicating that it is a homolog of gC1qR. To verify whether the C1q-like motif (a segment of globular heads) of *Pc*CLL can interact with C1qR (gC1qR homolog) in crayfish, we performed immunofluorescence staining (observed under a confocal microscope), co-IP, and pull-down assays. We subsequently found that the C1q-like sequence of *Pc*CLL interacts with C1qR (Fig. 2C through J). Studies have shown that C1qR has multiple immune functions as a multiligand-binding protein (22, 43). In vertebrates, C1qR binds ligands to induce the production of inflammatory byproducts that exert immune effects and activate early immune defense responses via immune pathways such as the complement and kinin-kallikrein systems in response to invasion by external pathogens (44, 45). In contrast, studies on gC1qR in invertebrates such as crustaceans have focused on its role as a receptor to recognize external pathogens (22, 23). According to previous studies, *Sp*gC1qR can interact with WSSV VP28 (23). In the present study, C1qR responded to WSSV infection in crayfish and was able to inhibit WSSV replication by binding to VP28 (Fig. 3).

According to the current study, lectins can act as pattern recognition receptors to activate the corresponding signaling pathways and thus perform the corresponding immune functions (46). STAT and Dorsal are phosphorylated and then enter the cell nucleus to initiate the transcription of immune molecules (26, 41, 47). *Pc*CLL collaborates with C1qR to promote the nuclear translocation of Dorsal (Fig. 4). MSK2 kinase regulates the expression of zinc finger protein 708 (*Ls*ZN708) by phosphorylating Dorsal in *Laodelphax striatellus* (48). SRPKs are splicing factors of serine-arginine proteins and are reportedly involved in the phosphorylation of various proteins (29, 30). SRPK induces the subcellular localization of SF2/ASF by mediating their phosphorylation (49). During Ebola virus (EBOV) infection, EBOV VP30 activates primary transcription of EBOV through phosphorylation by the specific kinase SRPK1 (30). SRPK1 activates the NF-κB signaling pathway by regulating the phosphorylation of IKK (50). In our study, *Pc*CLL and C1qR regulated Dorsal (one motif of NF-κB) nuclear translocation in an SRPK-dependent manner (Fig. 5). Previous studies have demonstrated that recombinant proteins can enter cells and then activate intracellular signaling pathways (37, 51, 52). In this study, pull-down and co-IP assays have demonstrated interaction between CLL and C1qR (recombinant and native proteins). Confocal microscopy revealed that CLL, C1qR, and VP28 were located on the cell membrane and interacted with each other (CLL and C1qR, C1qR and VP28) (Fig. 1 to 3). When crayfish are infected with WSSV after injection of rCLL or rC1qR, the rCLL or rC1qR may interact with C1qR or CLL on the membrane or in cells, thereby activating SRPK and Dorsal through an unknown mechanism.

**Fig 7:**
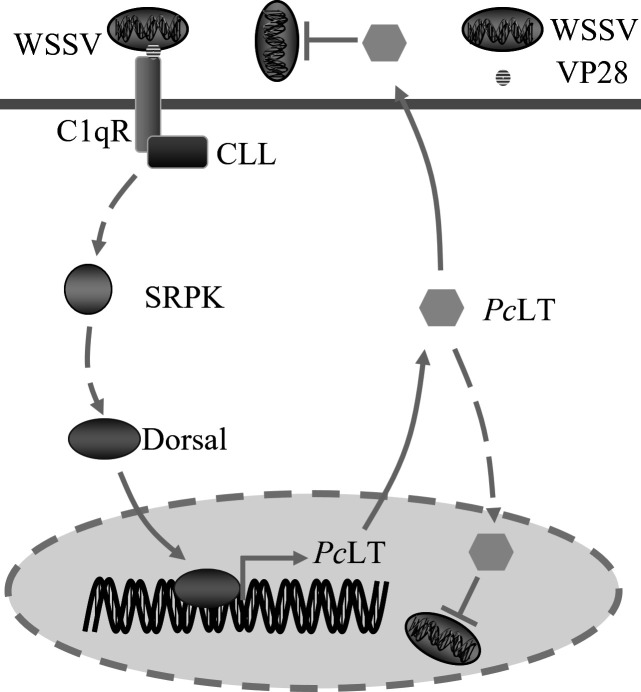
Molecular mechanism of *Pc*CLL and C1qR participation in the antiviral immune response. C1qR recognizes WSSV by interacting with VP28 (the main envelope protein of WSSV). Then, C1qR recruits *Pc*CLL and regulates the activation of Dorsal in an SRPK-dependent manner. SRPK promotes the activation and nuclear translocation of the transcription factor Dorsal by an unknown mechanism. After Dorsal enters the nucleus, it mediates the transcription of *Pc*LT, which is reported to be involved in the antiviral immune response.

Transcription factors translocate into the nucleus and regulate the expression of effector molecules to perform immune functions. *Pc*LT was reported to be involved in the anti-WSSV immune response (34). *Pc*CLL, C1qR, SRPK, and Dorsal all regulate the expression of the antiviral protein *Pc*LT (Fig. 6). The Dorsal binding site was found in the promoter sequence of the antiviral protein *Pc*LT (Fig. 6K and L). These results suggest that the *Pc*CLL-C1qR-SRPK axis inhibits WSSV reinfection by promoting Dorsal nuclear translocation to regulate *Pc*LT expression. In conclusion, as shown in Fig. 7, C1qR recognized WSSV through interaction with VP28 and subsequently recruited *Pc*CLL. C1qR and *Pc*CLL cooperate to promote the nuclear translocation of Dorsal. SRPK is an essential component in the nuclear translocation process of Dorsal. The C1qR*-Pc*CLL-Dorsal axis inhibited WSSV infection by regulating the expression of *Pc*LT (Fig. 7). In this study, we determined that *Pc*CLL and C1qR activate Dorsal in an SRPK-dependent manner, providing new insight into innate immunity.

## MATERIALS AND METHODS

### Animals and WSSV

Healthy red swamp crayfish were purchased from Weishan Lake, China. The animals were kept at 22°C for 1 week before the corresponding experiments were conducted. Crayfish with similar health status, body color, size, and weight (about 15 g each) were randomly assigned to the experimental group and the control group.

The WSSV strain previously stored in the laboratory was injected into crayfish, and when the red swamp crayfish were in a near-dead state, gill tissue (1 g) was collected and homogenized in 10 mL of phosphate-buffered saline (PBS, 140 mM NaCl, 2.7 mM KCl, 10 mM Na_2_HPO_4_, 2 mM KH_2_PO_4_, pH = 7.4) (51). The homogenate was then frozen and thawed twice at −80°C and centrifuged at 7,500 rpm for 15 min at 4°C. The resulting WSSV sample was stored at −80°C. For the infection assay, each crayfish was injected with approximately 5 × 10^5^ virus particles.

### Recombinant protein expression and antiserum preparation

Amplification of *Pc*CLL (NCBI Reference Sequence: XM_045738492.2) and C1qR (NCBI Reference Sequence: XM_045760562.2) was performed using EX-*Pc*CLL-F/R and EX-C1qR-F/R (Table 1). The PCR procedure was as follows: 95°C for 1 cycle for 5 min; 95°C for 35 cycles of 30 s; 58°C for 35 cycles of 30 s; 72°C for 35 cycles of 30 s; and 72°C for 1 cycle of 10 min. The amplified DNA fragments were subsequently ligated with pET-32a and pGEX-4T-1 at 22°C with T4 ligase (TaKaRa, Japan). The recombinant plasmid was transformed into BL21 (DE3) cells, and it was subsequently induced using isopropyl-beta-D-thiogalactopyranoside (IPTG). After that, purification was performed in the inclusion bodies of BL21 *Escherichia coli*. The purified recombinant proteins were used to immunize rabbits or mice to prepare the antiserum. Antibodies against VP24, VP26, VP28, β-actin, H3, GAPDH, Dorsal, and STAT were prepared in our laboratory (25, 52). His-Tag protein (a fusion protein containing 6×His expressed by pET-32a, about 12 kDa) and GST-Tag protein (a fusion protein expressed by pGEX-4T-1, about 25 kDa) were also prepared as subsequent controls.

**TABLE 1:** Sequences of the primers used in the study^*a*^

Primer	Sequence (5′−3′)
Recombinant expression	
EX*Pc*CLL-F	TACTCAGGATCCATGTTTGTGCTGGCGCTT
EX*Pc*CLL-R	TACTCACTCGAGTTTAAGAGAATGAACACC
EXC1q-like-F	TACTCAGGATCCGAGCCCTTCGTCACGATC
EXC1q-like-R	TACTCACTCGAGAGGCGTCAACTCGTTGGT
EXC1q-likeΔ-F	TACTCAGGATCCTTCTGGGTGGGTGCCTCT
EXC1q-likeΔ-R	TACTCACTCGAGTTTAAGAGAATGAACACC
EXC1qR-F	TACTCAGGATCCCTCTGCTCCAACAATCGC
EXC1qR-R	TACTCACTCGAGCTTGCGCTTCACAAAGTC
qRT-PCR	
RT18S-F	GCACCTGCTACCATCAAG
RT18S-R	GACTCGTCGTACTCCTCCTT
RT*Pc*CLL-F	AGACCTCCGTCCAAGAT
RT*Pc*CLL-R	CAGAAAGGCGTCAACTC
RTC1qR-F	CTTTGAAGTTGACCTACAGATTGGC
RTC1qR-R	ACTCGTTTGTGATACCTCGCTCCTC
RTSRPK-F	CGACGACGAGGAGCAGGAA
RTSRPK-R	GGAGAAGTGACCCCAGCC
RTDorsal-F	GGAGCAGCCACAAGCAAA
RTDorsal-R	ATCCACAGTTACGCACGA
RT*Pc*LT-F	TGTTAGGTTCCGTCTTGGTC
RT*Pc*LT-R	GTGTCTCGCAGAAGTATCCC
RT*Pc*lysi5-F	GGCTATGGGTTGTCATTCTG
RT*Pc*lysi5-R	ATGTAGTTGCGGATGGTCTT
RT*Pc*Thy-F	CGGAGAAACAAGCAAAA
RT*Pc*Thy-R	CATAGATGCCTTATCAAAAC
RT*Pc*PHB1-F	TGTCCTTCCTTCCATCACAAAT TCA CAA AT
RT*Pc*PHB1-R	GCCACCTGCTTCAACTCAAC
RTVP28-F	AGCTCCAACACCTCCTCCTTCA
RTVP28-R	TTACTCGGTCTCAGTGCCAGA
RNAi	
RNAi*Pc*CLL-F	**GCGTAATACGACTCACTATAGG**GACGTTCAGGCTGCGTCTGA
RNAi*Pc*CLL-R	**GCGTAATACGACTCACTATAGG**GTTGTGTAGTTAATAACC
RNAiC1qR-F	**GCGTAATACGACTCACTATAGG**TATCACAAACGAGTTTGCCG
RNAiC1qR-R	**GCGTAATACGACTCACTATAGG**GTCCAGGACA GTGTAAATTA
RNAiSRPK-F	**GCGTAATACGACTCACTATAGG**GTATGAAGCGACACAGTGGC
RNAiSRPK-R	**GCGTAATACGACTCACTATAGG**CCTCATATCAGGAGTAATCT
RNAiDorsal-F	**GCGTAATACGACTCACTATAGG**ACGCCCTGAAGCTAAGAGAG
RNAiDorsal-R	**GCGTAATACGACTCACTATAGG**ATCTTCTCTGTGTTTGCACT
ChIP	
ChIP*Pc*LT-F	CAAAACATTAAAACCGTC
ChIP*Pc*LT-R	CAGAAGCGGGGATTTA

^*a*^ Underlining indicates restriction enzyme sites, and bold font indicates T7 promoter sequences.

### Expression profiles

Detection and analysis were performed via qRT-PCR and Western blotting. For sample collection after virus injection, WSSV was injected into red swamp crayfish, and the control group was injected with an equal volume of PBS. RNA and protein were extracted from the gills of crayfish 24 h after injection. For qRT-PCR, 18S RNA was used as an internal reference. The qRT-PCR procedure was as follows: 94°C for 1 cycle for 2 min, followed by 40 cycles of 94°C for 15 s and 60°C for 30 s. The data were analyzed using the 2^-ΔΔCt^ method (53). For Western blotting, gills were homogenized in 1 mL of PBS, followed by centrifugation at 10,000 rpm for 12 min in a 4°C centrifuge, and the supernatant was collected. The protein samples were separated by 12% SDS-PAGE, transferred to nitrocellulose (NC) membranes, and then incubated with skim milk for 2 h. Primary antibodies were added, and the membranes were incubated overnight, washed three times with Tris-buffered saline with Tween (TBST), and then washed three times with Tris-buffered saline (TBS). Afterward, the membrane was incubated with horseradish peroxidase (HRP)-conjugated goat anti-rabbit IgG (BOSTER, China) or HRP-conjugated goat anti-mouse IgG (BOSTER, China) for 1 h and washed with TBST and TBS (54). The proteins were analyzed by chemiluminescence.

### RNA interference assay

The specific primers RNAi-*Pc*CLL-F/R, RNAi-C1qR-F/R, and RNAi-GFP-F/R (Table 1) were used in this assay. The dsRNA was synthesized using a T7 synthesis kit according to the manufacturer’s instructions, with specific gene fragments linked to the T7 promoter serving as the template (Thermo Fisher, USA). The dsRNA targeting the GFP gene was prepared in the same way as a negative control. The dsRNAs (30 µg) were injected into crayfish hemocoels. Forty-eight hours later, total RNA was extracted from the gills of the above crayfish to detect RNAi efficiency via qRT-PCR assays.

### WSSV inhibition assay

To determine the influence of *Pc*CLL and C1qR on WSSV replication, dsRNA (30 µg, targeting *Pc*CLL and C1qR) and recombinant protein (30 µg, rCLL and rC1qR) were applied. For the recombinant protein application assays, crayfish were infected with WSSV 2 h after rCLL or rC1qR injection. For the dsRNA application assays, crayfish were infected with WSSV 48 h after dsCLL or dsC1qR injection. Tag and dsGFP were used as controls. At 24 h post-WSSV infection, the mRNA, protein, and genomic DNA were extracted from gills of crayfish. The expression level of VP28 was determined by Western blotting, and the relative level of WSSV copies was determined by semiquantitative PCR assay. For the semiquantitative PCR assay to determine the relative level of WSSV copies, amplification was performed using genomic DNA as a template and specific primers for VP28 (listed in Table 1). The reaction was carried out with EasyTaq PCR SuperMix (TransGen Biotech, China) under the following conditions: 94°C for 5 min; 20 cycles of 94°C for 15 s, 55°C for 30 s and 72°C for 30 s; and a final 72°C for 10 min. The quantitative value of the destination strip = (total gray value of each destination band / total gray value of the internal reference band corresponding to each destination band) × 100%. The gray value of the control group was labeled as 1.0.

### Survival assay

The red swamp crayfish were divided into two groups of 30 animals each. For the recombinant protein application assays, crayfish were infected with WSSV 2 h after rCLL or Tag injection. The number of dead crayfish in each group was counted daily, and the survival rate of each group was calculated. For the post-RNAi survival verification assay, crayfish were infected with WSSV 48 h after dsRNA injection to determine their survival rate. A similar method was used for the survival assay of dsCLL-injected crayfish, as described above.

### Pull-down and co-IP assays

A pull-down assay was performed to verify whether *Pc*CLL and C1qR interact with each other, using rGST-*Pc*CLL and rHis-C1qR. First, the GST resin (Sangon, China) was washed with PBS five times, and 200 µg of GST-*Pc*CLL or 200 µg of GST-tagged protein were added separately and incubated for 30 min at 4°C, followed by washing with PBS five times. Then, 200 µg His-C1qR was added, and the mixture was incubated for 2 h at 4°C and then washed with PBS five times. The GST resin was eluted with elution buffer (50 mM Tris-HCl, 10 mM reduced glutathione, pH 8.0); the samples were analyzed by SDS-PAGE, stained with Coomassie Brilliant Blue, and the protein signals were analyzed by imaging. A pull-down assay was also performed to detect the interaction of His-C1q-like, His-C1q-likeΔ, and His-VP28 with GST-C1qR.

Pull-down and Western blotting assays were used to analyze the interaction between WSSV envelope proteins (VP24, VP26, and VP28) and GST-C1qR. GST-C1qR (200 µg) was incubated with GST resin for 30 min and then washed with 5 mL of PBS. Approximately 500 µL (4 µg/µL) of gill homogenate from crayfish (after WSSV infection for 48 h) was added to the tube and incubated for 2 h at 4°C. Each tube was subsequently washed with PBS five times, and the GST resin was eluted with elution buffer. The eluted sample was analyzed by Western blotting.

Co-IP assays were performed to detect the interaction of C1qR with either CLL or VP28. Protein A resin (20 µL, Sangon, China) was washed with PBS five times. Antiserum (2 µL, anti-C1qR, anti-CLL, or anti-VP28) was added to each tube. Each tube was subsequently washed with PBS three times, and 1 mL of gill homogenate from crayfish (after WSSV infection for 48 h) was added to each tube and incubated for 2 h at 4°C. After being washed with PBS five times, the prepared samples were detected via Western blot.

### Immunofluorescence staining

To determine the influence of *Pc*CLL and C1qR on nuclear translocation of Dorsal, dsRNA (30 µg, targeting *Pc*CLL, C1qR, and SRPK) and recombinant protein (30 µg, rCLL and rC1qR) were applied. For the recombinant protein application assays, crayfish were infected with WSSV 2 h after rCLL or rC1qR injection. For the dsRNA application assays, crayfish were infected with WSSV 48 h after dsCLL, dsC1qR, or dsSRPK injection. Tag and dsGFP were used as control. At 6 h post-WSSV infection, the hemolymph was collected using a syringe containing a mixture of anticoagulant (0.14 M NaCl, 0.1 M glucose, 30 mM trisodium citrate, 26 mM citric acid, and 10 mM ethylenediaminetetraacetic acid, pH 4.6) and 4% paraformaldehyde and centrifuged at 2,000 rpm for 5 min. Afterward, the hemocytes were resuspended in PBS and added dropwise to slides coated with poly-L-lysine. Then, 1% Triton dissolved in PBS was added dropwise onto the slides. After being washed three times with PBS, the slides were blocked with 1% bovine serum albumin (BSA) for 1 h at 4°C. Anti-Dorsal antibody (1:100 dissolved in 1% BSA) was added, and the samples were incubated overnight at 4°C. After washing three times with PBS, goat anti-rabbit Alexa Fluor 488 (1:200 dissolved in 1% BSA, Beyotime, China) was added, and the mixture was incubated for 2 h in a dark room temperature environment. After being washed five times with PBS, 4′,6-diamidino-2-phenylindole (DAPI) (Sigma, USA) was added, and the cells were incubated at room temperature, washed six times with PBS, and observed under a confocal microscope.

An immunofluorescence staining assay was performed to detect the colocalization of CLL with cell membranes. The hemocyte membranes (red) were stained with a Cell Plasma Membrane Staining Kit with Dil (Beyotime, China). Hemocytes were collected 3 h after WSSV infection in crayfish. Subsequent experiments were performed as described above. The colocalization of C1qR and CLL was analyzed. Anti-C1qR mouse serum and anti-CLL rabbit serum were used in this assay. Hemocytes were collected 3 h after WSSV infection in crayfish and then fixed with 4% paraformaldehyde and permeabilized with Triton. After the samples were blocked with BSA, primary antibodies against C1qR or CLL were added, and the samples were incubated overnight at 4°C. After being washed with PBS, goat anti-mouse Alexa Fluor 594 (Beyotime, China) and goat anti-rabbit Alexa Fluor 488 were added, and the samples were then stained with DAPI. The colocalization of C1qR and VP28 was also detected via confocal microscope using anti-C1qR mouse serum and fluorescein isothiocyanate (FITC)-labeled VP28 rabbit serum (Sigma, USA).

### Separation of nuclear, cytoplasmic, and membrane proteins

A nuclear protein extraction kit (Beyotime, China) was used. Six hours after crayfish were infected with WSSV, the gills were added to a mixture of cytoplasmic protein extracts A and B containing phenylmethanesulfonyl fluoride (PMSF) (1 mM final concentration), followed by homogenization. Samples were centrifuged at 1,500 × *g* and 4℃ for 5 min, and the supernatant contained the cytoplasmic protein (6 mg). The precipitate was resuspended in cytoplasmic protein extract A and centrifuged at 10,000 rpm and 4℃ for 5 min. Then, the precipitate was resuspended in nuclear protein extract, vortexed, and incubated on ice for 30 min. The samples were centrifuged at 10,000 rpm and 4℃ for 10 min. The resulting supernatant contained nuclear protein (0.2 mg).

A membrane and cytosol protein extraction kit (Beyotime, China) was used following the manufacturer’s instructions. The proteins were collected 3 h after infection with WSSV. Membrane protein (0.5 mg) and cytosolic protein (20 mg) were obtained. Because there is no commercial reference antibody for crustacean membrane proteins, the membrane protein samples were analyzed using SDS-PAGE (stained with Coomassie Brilliant Blue) as a loading control for the membrane proteins.

### Chromatin immunoprecipitation

The ChIP assay was performed according to previously described methods (55, 56). Crayfish were injected with WSSV. The gills were collected 6 h after WSSV infection and used as the pool for ChIP. The primers used for amplifying the *Pc*LT promoter are listed in Table 1.

## Data Availability

Raw data files for all figures are available in Files S1 and S2 in the supplemental material.

## SUPPLEMENTAL MATERIAL

File S1 jvi.00416-25-s0001.xlsx Original images of Fig. 1 to 4.

File S2 jvi.00416-25-s0002.xlsx Original images of Fig. 5 and 6 and qRT-PCR, survival, and immunofluorescence intensity statistics for Fig. 1 to 6.

Figure S1 jvi.00416-25-s0003.docx Nucleotide sequences and amino acid sequences of *Pc*CLL.

Supplemental legends jvi.00416-25-s0004.docx Legends for Files S1 and S2.

ASM does not own the copyrights to Supplemental Material that may be linked to, or accessed through, an article. The authors have granted ASM a non-exclusive, world-wide license to publish the Supplemental Material files. Please contact the corresponding author directly for reuse.
