# Supplementary material for: CLL, together with C1qR, suppresses WSSV infection by regulating the activation of Dorsal
Source: J Virol. 2025 Oct 13;99(11):e00416-25. doi: 10.1128/jvi.00416-25 (PMC12645952; doi:10.1128/jvi.00416-25)
Supplement: Figure S1 — Nucleotide sequences and amino acid sequences of PcCLL. [file jvi.00416-25-s0003.docx]

We submit the raw data in two files (JVI00416-25-S1 and JVI00416-25-S2).

**JVI00416-25-S1** file contains all original images of Fig 1, 2, 3 and 4.

**JVI00416-25-S2** file contains all original images of Fig 5 and 6, and all qRT-PCR, survival and immunofluorescence intensity statistics for Fig 1, 2, 3, 4, 5 and 6
