# Supplementary material for: CLL, together with C1qR, suppresses WSSV infection by regulating the activation of Dorsal
Source: J Virol. 2025 Oct 13;99(11):e00416-25. doi: 10.1128/jvi.00416-25 (PMC12645952; doi:10.1128/jvi.00416-25)
Supplement: Supplemental legends — Legends for Files S1 and S2. [file jvi.00416-25-s0004.docx]

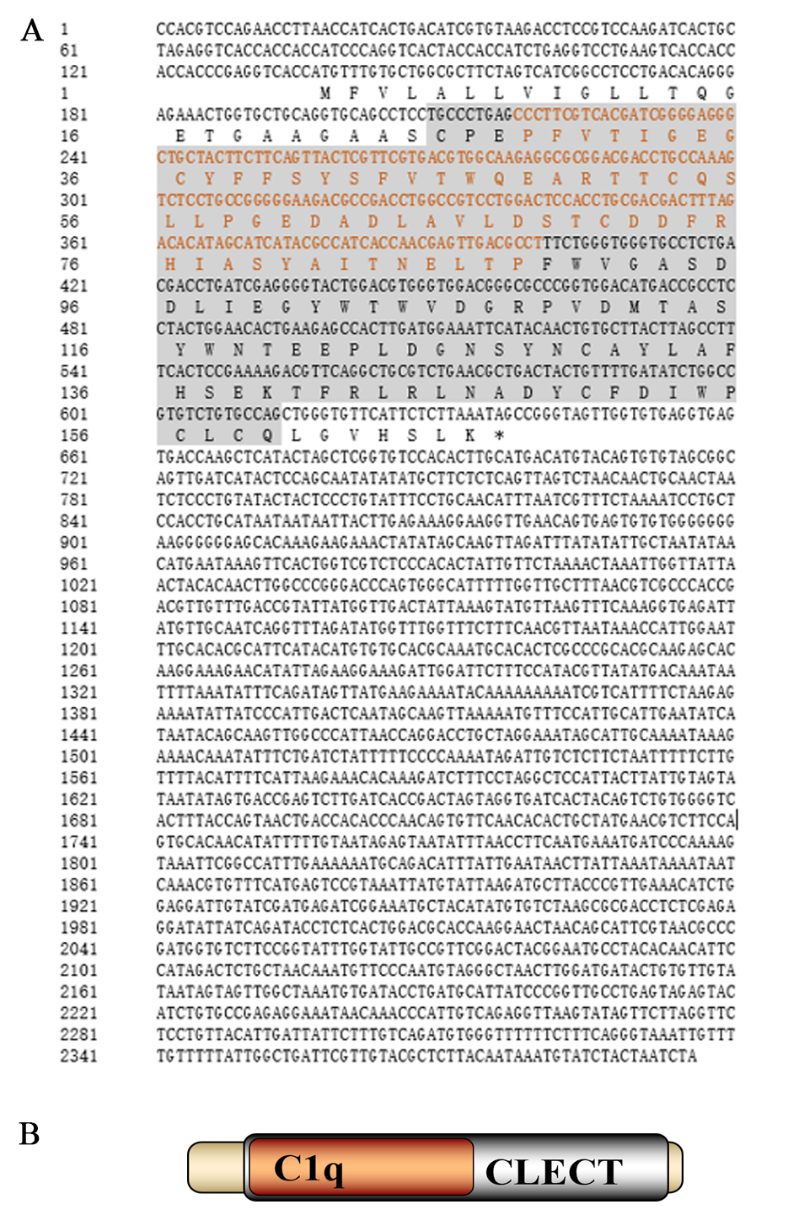


**Fig. S1. Nucleotide sequences and amino acid sequences of *Pc*CLL.** (A) The hatched section shows the CLECT domain and the orange section shows the C1q-like motif. (B) Structural analysis of the amino acid sequences of CLL was performed using SMART online website.
